# Leadership of education psychological services: fit for purpose?

**DOI:** 10.1080/02667363.2013.808606

**Published:** 2013-06-18

**Authors:** Roger Booker

**Affiliations:** Educational Psychology Group, Department of Clinical, Educational and Health Psychology, University College London, London, UK

**Keywords:** leadership, psychological services, leadership capacity, authentic leadership, systems thinking

## Abstract

At a time of great change for educational psychology services in England, this paper reviews current theories of leadership and proposes how an integration of key aspects of these can be applied to support a self review of leadership practice, both by individual leaders and by services. The message from current theory is that in the midst of complexity and rapid change, a primary focus on the head of service is outdated; there should instead be a focus to develop the leadership capacity of the service as a whole. Key constructs considered are systems thinking, social identity, authenticity, and leadership as social construction.

## Introduction

It is incontrovertible that educational psychology services in England are being required to examine critically their service delivery model in the wake of public spending cuts. The most common response has been a shift towards a greater trading of services with schools and other bodies, often with pleasing results in terms of increased demand for services and an acceptance that the skills of psychologists are worth paying for. Alongside this there may have been a broadening of the client base (organisational and individual) with a widening of the working context for many educational psychologists (EPs). The potential context for EP work is therefore expanding. A corollary to this is that services are going to have to be more dynamic, entrepreneurial and alert to feedback.

While there has been some discussion about the opportunities these changes have offered, and the ethical dilemmas posed, there has been little discussion about the importance of leadership of services as they confront these challenges. This paper intends to initiate such a discussion and provide a basis for self evaluation and development through a review of current theories of leadership, followed by discussion of how those that are most congruent with the discipline of psychology might be incorporated into the culture of psychology services at the present time. It draws on the review in Booker (2012) which addresses leadership in children's services.

When working with psychologists as part of a leadership and management development programme it has repeatedly been the author's experience that there is confusion between “leadership” and “management” so the paper starts by clarifying this distinction. The paper then reviews some current theories of leadership, proceeds to discuss their application to leadership roles at all levels of a psychology service and concludes by examining the training and development implications.

## Distinctions between leadership and management

Day (2000) has identified management as the application of proven solutions to known problems involving the implementation of “standard operating procedures”: from this perspective managers require specific knowledge, abilities and skills to enhance task performance in management roles – this performance focusing on regulatory processes such as monitoring, target setting, performance management of individuals, and financial control. Management produces consistency and order; leadership produces movement (Kotter, 1990). Leadership implies the creation and articulation of a vision for the organisation or team. The leader, as an individual, embeds and transmits the organisation's culture and values (Schein, 1985). Leaders are at the forefront when groups need to learn their way out of problems that could not have been predicted; they take key decisions at times of difficulty or crisis which determine future direction; and they foster collaboration and build trust so as to enable others in the organisation to act.

However the distinction between “managers” and “leaders” applied differentially to roles across an organisation has been questioned (Bolden, 2004). Any individual in a role of formal management authority is both a manager *and* a leader – particular acts usually embodying both functions simultaneously. For example, an evaluation of service delivery to a confederation of schools will review the objectives agreed for the year and the perceived outcomes (an act of management), and negotiate future service in the light of changing priorities within the confederation and the psychology service. This requires a strategic understanding beyond the immediate context, imaginative re-working of service delivery, and skills of persuasion and negotiation – all requirements of leadership.

## Contexts for leadership in a psychology service

Psychology services operate in diverse political and organisational contexts. Some have experienced comparatively little change in response to recent government policy; others have found themselves displaced into new divisions within a Children's Service, with a revised brief and radically reduced core budget. These changes are a response to the changes that Children's Services have been required to make which are as diverse as the response earlier to the previous government's initiatives around integrated services (Booker, 2005). Additionally, services are managing change to their internal working context with the entry of newly trained, doctorate level staff, together with experienced staff who have completed a continuing professional development (CPD) doctorate; experiences which may have raised expectations of professional practice both in terms of the range of activity undertaken and the quality of the practice base. Newly trained EPs are predominantly of the Millennial Generation (Balda & Mora, 2011) and bring new approaches to relationships and knowledge which are out of kilter with many aspects of conventional leadership practice.

The challenges that services face as a consequence of this include all or some of the following:

- To maintain service delivery alongside staff redundancies (or threat of), which includes interim acting up management and leadership
- To enhance service profile within revised children's service structures through constructive and assertive engagement
- To meet the expectations of more highly trained and developed staff
- To manage an external tension between those who respond to traded demands and those that offer a funded “core” service or an internal tension when these are both undertaken by single individuals
- To develop new partnerships with allied agencies
- To establish links with not for profit organisations in a shift towards a community psychology orientation
- To build on the skills of multi-professional working demanded by the coalition government's revised approach to the management of special educational needs.

## Current theories of leadership

The various strands of research into leadership offer perspectives that complement and overlap rather than compete with each other. They also draw more on psychological theory than any other discipline. The following strands have been identified from reviews by van Knippenberg, van Knippenberg, De Kremer, and Hogg (2004), Avolio, Walumbwa, and Weber (2009), Bolden (2004), and Bennett, Wise, Woods, and Harvey (2003) as having particular relevance. These strands are as follows:

- Charismatic/transformational leadership
- Distributed leadership
- Complexity leadership
- Authentic leadership
- Identity based leadership
- Leadership as socially constructed

What follows is a summary of each theory: not all are concerned with “effectiveness”, evidence for which is notoriously difficult to establish since criteria for effectiveness are themselves differently defined according to the aims and values of the organisation concerned. Some focus on the processes of leadership in contemporary organisations and identify different ways of understanding leadership. These are presented without specific reference to psychology services, these implications being addressed subsequently.

### Charismatic and transformational leadership

Charismatic and transformational models developed out of the traditional notions of leadership that prevailed during the last century. According to Bass, 1985 (cited by Liao & Chuang, 2007), transformational leaders display four types of behaviours which enable followers to transcend self interest and perform beyond expectations. *Charisma* leads to trust and identification with the leader; *inspirational motivation* derives from the articulation of a compelling vision; *intellectual stimulation* encourages followers to challenge assumptions and take risks; *individualised consideration* treats followers on a one to one basis (Liao & Chuang, 2007). These leader characteristics have been found to correlate positively with organisational outcomes across many different types of organisations and situations (Judge & Piccolo, 2004). However Bolden (2004) notes that a tendency for charismatic leaders to desert their organisations after making their changes, leaving behind unresolved challenges, can result in this form of leadership being unsustainable in the long term. Lawler (2007) argues that the focus of attention on the individual leader in the public sector has been taken at the expense of a more considered examination of the relevance of distributed leadership.

### Distributed leadership

The idea of distributed leadership derives from understanding leadership as a process of influence that can be exercised by any member of a group, regardless of formal position of authority. Bennett et al. (2003), on the basis of an exhaustive literature review, identify three distinctive elements:

- Leadership as an emergent property of a group or network of interacting individuals
- An organisational openness to loosening the boundaries of leadership
- An understanding that varieties of expertise are distributed across the many and not the few

Openness to loosening the boundaries of leadership potentially leads to a tension with a concern by central leadership to retain control and direction. The internal and external culture of the organisation will be important influences on the extent to which distributed leadership can develop. Carson, Tesluk, and Marrone (2007) suggest that the organisational climate should be characterised by shared purpose, an emphasis on social support within teams, and an expectation that all team members should have a voice. For those in formal leadership roles there can be significant emotional challenges in dismantling the construct of the charismatic leader, strongly held by many chief executive officers (CEOs) (Huffington, James, & Armstrong, 2004).

### Complexity leadership

Complexity theory views organisations as non-linear systems with disorderly dynamics where outcomes are only partly predictable and often unexpected (Stacey, 1995). Leadership in complex systems is viewed as “an interactive system of dynamic, unpredictable agents that interact in complex feedback networks, which can then produce adaptive outcomes such as knowledge dissemination, learning, innovation, and further adaptation to change” (Uhl Bien, Marion, & McKelvey, 2007). Rather than a focus on individuals in formal positions of authority in an organisation, complexity leadership focuses on the dynamic, complex *systems and processes* that comprise leadership and which occur throughout the organisation while adapting to new challenges. This process is referred to as *adaptive leadership.*

In the context of bureaucratic organisations it is envisaged that there are two other kinds of leadership. The first is *administrative leadership*, the actions of individuals or groups in formal management roles who plan and organise activities to accomplish organisationally prescribed outcomes. The second is *enabling leadership*, which works to catalyse the conditions in which adaptive leadership can thrive. These three leadership functions are intertwined (or *entangled)* and can help or oppose one another. Complexity leadership extends the ideas of distributed leadership with a stronger focus on creating new patterns of activity, learning and adapting to rapidly changing environments.

The challenge in complexity leadership is to determine how organisations enable and coordinate these dynamics without suppressing their adaptive and creative capacity. When administrative leadership opposes adaptive leadership it thwarts it with overly authoritarian or bureaucratic control structures. Enabling leadership serves primarily to enable effective adaptive leadership through tailoring the behaviours of the other two so that they can function in tandem with one another.

### Authentic leadership

The idea of authentic leadership is rooted in a *relational* understanding of leadership as involving not just the individual leader but their follower(s) and the context within which they operate. Avolio et al. (2009) suggest that there are four components to authentic leadership: *balanced processing* (the dispassionate analysis of relevant data), an *internalised moral perspective, relational transparency* (openly sharing information and feelings as appropriate), and *self awareness.*

For Goffee and Jones (2006) there are three critical elements: a consistency between words and deeds, coherence in role performance, and comfort with self. These complement each other in an authentic leadership performance: doing what you say (“walking the talk”), this “doing” having a consistent underlying thread across different contexts and emerging from an individual with sufficient security in themselves as a person to inhabit the role in a way that is clearly their own. The key is to “be yourself more, with skill” (Goffee & Jones, 2006). As the differences in these two definitions imply there is no agreed definition of “authenticity”. In itself it does not imply ethical or behavioural integrity, although proponents assume this. Crucially, authenticity is a quality attributed to a leader by followers.

### Identity based leadership

The point of departure for identity based leadership theory is that effective leadership cannot be understood without a focus on the psychological effects that the leader has on her or his followers. Starting from the premise that the way in which we perceive ourselves, our self concept or identity, has profound effects on the way in which we feel, think and behave, research has investigated ways in which leadership can change follower identity (van Knippenberg et al., 2004).

It is argued that if leadership is able to influence and develop follower identity, changes will feed into follower motivation, attitudes and behaviour. This is of great significance because motivation that flows from self conception is intrinsic to the individual and less contingent on monitoring and external reward (Ryan & Deci, 2000). When the (organisational) self is defined in collective terms, *collective* interest is experienced as *self* interest and individuals are intrinsically motivated to contribute to the collective good. Also important is the leader's impact on a follower's relational self concept. Leader behaviour which results in a follower personally identifying with the leader will engender trust in the leader and increased commitment and motivation.

To adopt the phrase of Reicher, Haslam, and Hopkins (2005), leaders have to become *entrepreneurs of social identity*, seeking to facilitate the emergence of an inclusive identity for the group which embraces all members and whose values and priorities are realised in the aims and actions they propose.

### Leadership as social construction

Social constructionist perspectives (Campbell, 2000; Gergen, 1999) have influenced thinking about how leaders make sense of their own reality and how followers make sense of the leader. At an organisational level there will be a number of “discourses” regarding leadership, followership and the organisation's relations with its environment.

Shamir (2007) has argued that many leadership theories can give the impression of the follower as a rather passive recipient of influence. An alternative view, originally presented by Meindl (1995), assumes that the relationship between leaders and followers is primarily a constructed one (by followers), which is heavily influenced by follower context and relationships. It shifts the emphasis away from the substantive significance of the leader's behaviour or personality. It assumes that followers react to, and are more influenced by, their *construction* of the leader than they are by any “true” assessment of the individual concerned. A fundamental assumption is that followers have between them a range of multiple realities through which the leader is viewed by the follower as taking a position in relation to one or more of the leadership discourses available within the organisation (Campbell & Groenbaek, 2006).

## Integrated perspectives on leadership?

This brief review indicates that leadership theories have many common features, and in some instances can be considered to complement each other. The most significant elements from the earlier theories are:

- a shift away from the exclusive focus on the leader at the top of the organisation to one of leadership at all levels; leadership that can *emerge* as well as being formally assigned
- a realisation that alongside leadership there is a need to re-evaluate the way in which organisations and the environments they operate in are becoming increasingly complex to the extent that hierarchical management and leadership can no longer hope to exercise the degree of control they have done in the past
- a focus on followers and the processes through which they perceive leadership, and the influence this has upon their understanding of themselves in their organisational role
- in addition to the well known aspects of charismatic leadership, a focus on *how* the leader (at any level of the organisation) interacts with staff through the expression of an authentic self
- a greater awareness of specific organisational context and how it is socially constructed by both the leadership and by members

From the perspective of an applied psychologist, this represents a strongly systemic perspective on leadership, one that considers it to be essentially relational, dependent on internal and external context, operating at all levels and contingent on the differing realities of leaders and followers. However, a more traditional view which focuses on charismatic and transformational leadership remains the dominant “theory in use” in many organisations – particularly in the public sector – where context is almost entirely neglected (Peck, Freeman, Perri 6, & Dickinson, 2009). While contemporary research still regards the behaviour and characteristics of the leader at the top of the organisation as important, this role is placed in a much more differentiated context of the organisation as a whole. It is one where rapid and innovative responses are needed at the interfaces of the organisation in response to a fast changing environment, where information is far more widely available to service users. These external conditions render the older models of predominantly top down leadership no longer fit for purpose.

## Implications for leadership in a psychology service

Some implications for psychologists at different levels of authority in a service are now considered. No single leadership theory predominates in this discussion; all are referred to at relevant points. Underpinning the whole discussion is an assumption of a capacity for systemic thinking.

### The principal educational psychologist (PEP)

These comments apply equally to management senior EPs as to principal educational psychologists (PEPs). While concern about too great a focus on the top down charismatic model for the head of an organisation may be a valid point in relation to large organisations such as children's services, it applies less to smaller ones such as psychology services where a PEP's engagement with staff tends to be relatively frequent, and within a culture where professional autonomy is emphasised, communication more open and feedback on service delivery expected. Nevertheless, even in this more intimate context, the “charismatic” head of service needs to be alert to the risks of detachment from the rest of the service on the one hand, or of creating dependency on the other hand. The size and primary task of psychology services, leading to a strongly relational culture, would argue strongly for authenticity to be a prime concern for the head of service; second to this an ability to read the changing context within which the service is located, and finally to be alert to and encourage emergent leadership, formal and informal, across the service.

Authenticity implies for the head of service, firstly, the discovery about what it is inside themselves that they can mobilise in the leadership context (Goffee & Jones, 2006). This mobilisation lends a uniqueness to the way the leader takes up the role and makes it their own and not some “clone” of what a good leader is thought to be. On display are a particular set of personal characteristics and values that “work” for this particular individual through day to day interaction and which enables EPs to identify with the leader and reap the benefits of self motivation which is part of the identification theory of leadership. This leads to marked individual differences across leaders in terms of style and personality. Compare, for example, Angela Merkel, Richard Branson and Jose Mourinho. These leaders, consciously or otherwise, have worked out what it is about themselves that they can deploy effectively as leaders and we do, in each case, have a sense of “knowing” them (without necessarily wanting to adopt them as specific role models). So confidence and security in those aspects of ourselves that we deploy are essential, bringing the necessary personal authority to the role.

Current leadership theory emphasises the relatedness of leadership and the importance of understanding and managing the context. This is a crucial skill for the head of service, and in particular in a climate of retrenchment where powerful defensive feelings arise. Observation, reflection and judgement are key processes here which need to supersede task focus and action. This is best done in consultation with trusted colleagues who can maximise the amount of available information from the organisation and enable a service to shape the context rather than just be in the position of reacting to it. In this way new opportunities can be identified. Interestingly, judgement is now regarded as the key leadership skill at INSEAD (European Institute of Business Administration) (Pant, 2012).

A further implication for adaptive leadership at the head of service level is that there is a role to create disequilibrium (Tetenbaum & Laurence, 2011). In a time of turbulent change this will most likely take the form of transmitting disequilibrium from the environment and clarifying for the service what the implications of change are, identifying the challenges and then passing the problem over to the service for solutions. Such an approach follows from an expectation that leadership lies across the service and not solely with the formal authority of the leader. However, careful judgement is needed here to ensure that the PEP retains enough of their “containing” function for staff uncertainty and anxiety and that they are not left foundering.

The key message, then, from current leadership theory is that alongside the formal leader roles leadership should emerge at all levels in the organisation, in particular where environments are dynamic and the head of the service cannot possibly keep in touch with the multiple interfaces with which EPs engage. Particular emphasis is placed upon front line staff as a context for leadership. This is where “the moment of truth” (Normann, 1984) lies: the crucial, skilled and informed interactions with vulnerable children, families and other professionals which aim to bring about positive change and which ultimately determine the effectiveness of the service. It is salutary for a head of service to contemplate the number of such moments of truth engaged in by their EPs over a single day, each one of which should be a representation of professional expertise and service reputation enhancement. It is therefore crucial that the head of service should see leadership as essentially enabling so that there is a strong downwards accountability to staff as well as an upwards one to service line management. This aligns well with proposed expectations of leadership from the Millennial generation (Balda & Mora, 2011) who expect communication to be open, positive and in the form of a dialogue, hierarchy to be flat, and relationships to be built on the basis of trust rather than authority.

### Front line staff

There are two requirements for emergent leadership: a culture generated by those in formal positions of authority that views leadership as adaptive, distributed and not restricted to their roles alone, and an enabling and confidence in staff across the service to exercise leadership. This might require a rehearsal of what leadership means in different contexts as well as explicit permission and clarity regarding accountability. This particularly applies to services where an entrepreneural approach is developing in the context of traded services. Within the service there will then be a unique discourse on leadership with which all staff can identify.

Those not in formal positions of authority can take up leadership roles as part of their service delivery and/or within the service itself. It is not uncommon for the latter to happen when an individual chooses (or is asked) to lead on a particular initiative. Responsibilities for different aspects of a particular project may be shared across a team. These are common experiences and a key element of a leadership culture within a service. Some of the heightened expectations of new entrants and those who have gained CPD doctorates can be met in this way.

In terms of service delivery, a particular challenge arises in the context of multi-professional working, in particular when part of a multi-professional team. The requirements of team working have now become familiar: a willingness to engage, collaborate and learn; a sign up to the aims of the team; an extension of role understanding and identity to embrace these aims; clarity about one's professional role and its overlap and distinctiveness in relation to other professional roles; respect for and valuing of difference; ability to manage and resolve tension; and ability to operate in new ways (see for example Anning, Cottrell, Frost, Green, & Robinson, 2006; Daniels, Leadbetter, & Warmington, 2007; Leadbetter, 2006; Watson, 2006). The “team around the child” approach has extended this vision to all case-work whether or not those involved are part of a formally constituted team, and retains its relevance in the context of new changes to addressing the needs of vulnerable children contained in the Children and Families Bill (http://www.education.gov.uk/a00221161/children-families-bill). Psychologists are often the professionals with these skills and a precursor to exercising them is a confidence and understanding of the leadership required.

### Leadership capacity

This emphasis on leadership at all levels strongly suggests that we should be thinking in terms of service *leadership capacity* (Day, 2000): one that maintains the distinction between administrative and adaptive leadership found in complexity theory. For those in formal authority the implication is to enable adaptive leadership throughout the service and to manage any perceived loss of control that this brings. For others the implication is to develop their role understanding to include initiative taking, making new professional liaisons and communicating effectively with all interfaces with the service about what it can offer. For this to be maximally effective, services will need to ensure that efficient feedback mechanisms are in place so that all members of the service are familiar with each other's initiative, as well as a commonly developed framework for development which acts as a container for a potentially wide range of activity. This feedback must relate not just to information but also to *knowledge* management so that what is brought into the service by newly trained and doctorate programme staff and within service projects feeds into universal practice.

Such a service-wide understanding would arguably build organisational resilience and enhance capacity to manage instability; it should also have the impact of enhancing service profile and establishing new liaisons with other organisations in order to operate more effectively in a market for services.

## Implications for training and development

If the earlier mentioned analysis of leadership functions is accepted there are significant implications for training and development within psychology services.

The first implication is for the service as a whole, which might usefully examine its own construction of leadership and the responsibility of all members to exercise leadership in the light of current theory and the specific challenges faced. While this might usefully be facilitated externally there is no necessary requirement for this if a planning process is distributed across all levels of the service and there is a commitment to a reflective input from all members regarding their own roles.

Core professional training makes very little of the EP as leader. In the writer's experience it can be helpful to reflect with a trainee group on their feelings about leadership and to consider what might be the first steps for them in exercising initiative and leading on an issue, especially in the third year of training. Across the curriculum there are at least three areas where greater emphasis could be laid on leadership: firstly, in seminars on multi-professional working where the focus otherwise tends to be on the maintenance of productive relationships and team dynamics; secondly, a trainee is frequently asked to undertake an appraisal of a service where they are doing a placement. How often is there a requirement to look at the service from a leadership perspective? Finally, in their own working groups on the training course there could be a requirement to reflect on leadership roles and how they were enacted, with feedback to individuals from the group.

For those in formal positions of authority the optimal way of addressing leadership issues is through a coaching relationship; however, given that this might be difficult to fund or organise (although peer arrangements can be highly productive), there are a number of questions which an individual leader might reflect on:

(1) What aspects of my personal self am I bringing into my role as a leader? How do these aspects appear to “come together” in the minds of members of the service and others outside in such a way as to enhance my effectiveness?
(2) What qualities *don't* I possess and how am I enabling others to ensure that these are addressed?
(3) In the midst of huge change do I communicate a compelling vision for the service with which everyone can identify? Can I consider myself to be an entrepreneur of social identity?
(4) How ready am I to see leadership exercised at all levels of the service and how do I enable every member to do this?
(5) How well am I reading the context and connecting with key external players?
(6) How equipped am I to apply a systemic understanding to the service and its context and how do I engage with other senior managers to facilitate this and make decisions?
(7) Have I established the best balance between containing the anxieties of the service regarding uncertainty and change on the one hand and communicating the urgency of reviewing and adapting practice on the other hand?

It is not just number (6) which is facilitated by a systemic approach to leadership – all of the questions can be considered to be systemic questions which emphasise interactions within a specific context and a social constructionist perspective of multiple realities (Campbell, 2000; Campbell, Caldicott, & Kinsella, 1994). Within local authority cultures, which tend towards linear cause and effect and immediate solutions to problems, it is a challenge to maintain this perspective. One option is to engage in experiential work in a secure development environment with colleagues who share a common set of leadership challenges and where the focus is on exploring dilemmas and the context within which they arise. For example, a head of service may have a dilemma of how to maintain motivation in staff against a background of budget cuts and re-organisation. While knowledge of systems thinking, of motivational theory and “leadership” will be a necessary backdrop to any response, the here and now dilemma is best addressed through an exploration of how such ideas fit within this specific service, its history and the meanings for individual members. Such an exploration can only take place through questioning which recognises that the head of service is dealing with processes that are not simple cause and effect ones, but ones which are essentially interactional and circular, with multiple levels of meaning and with complex ramifications that cannot be worked out in advance. The learning arises not from finding a “solution” but at two levels: firstly a set of possible leadership actions which address, in this example, the motivational issues for the team and, more importantly, a capacity to make sense of future dilemmas in a far more sophisticated way.

## Conclusion

This paper has reviewed recent developments in leadership research and has found a strong shift away from a focus on those at the top of an organisation. As a consequence of the need to respond to fast changing environments and for clients to have a much greater influence on the nature of the service provided, leadership is considered as an emergent function within psychology services: displayed not just by those in formal authority but in those at the front line of service delivery. Services should consider their *leadership capacity* at all levels and commit development resources for leadership accordingly. There are particular challenges for heads of service to show authenticity, to observe and “read” the service context before making judgements, and to enable adaptive leadership.
